# Biochemical and genetic characterization of *Trypanosoma cruzi N*-myristoyltransferase

**DOI:** 10.1042/BJ20131033

**Published:** 2014-03-28

**Authors:** Adam J. Roberts, Leah S. Torrie, Susan Wyllie, Alan H. Fairlamb

**Affiliations:** *Division of Biological Chemistry and Drug Discovery, College of Life Sciences, University of Dundee, Dundee DD1 5EH, U.K.

**Keywords:** Chagas’ disease, click chemistry, drug target, N-myristoylation, *Trypanosoma cruzi*, validation, CAP5.5, cytoskeleton-associated protein 5.5, DIG, digoxigenin, DKO, double knockout, DMEM, Dulbecco’s modified Eagle’s medium, HYG, hygromycin phosphotransferase, NMT, *N*-myristoyltransferase, NMT^OE^, NMT overexpressor, PAC, puromycin *N*-acetyltransferase, RTH/FBS, RPMI 1640 medium supplemented with trypticase, haemin, Hepes and 10% heat-inactivated FBS, SKO, single knockout, *Tb*NMT, *Trypanosoma brucei* NMT, TCEP, tris-(2-carboxyethyl)phosphine, *Tc*NMT, *Trypanosoma cruzi* NMT, *Tc*TryR, *Trypanosoma cruzi* trypanothione reductase, WT, wild-type

## Abstract

Co- and post-translational N-myristoylation is known to play a role in the correct subcellular localization of specific proteins in eukaryotes. The enzyme that catalyses this reaction, NMT (*N-*myristoyltransferase), has been pharmacologically validated as a drug target in the African trypanosome, *Trypanosoma brucei*. In the present study, we evaluate NMT as a potential drug target in *Trypanosoma cruzi*, the causative agent of Chagas’ disease, using chemical and genetic approaches. Replacement of both allelic copies of *TcNMT* (*T. cruzi* NMT) was only possible in the presence of a constitutively expressed ectopic copy of the gene, indicating that this gene is essential for survival of *T. cruzi* epimastigotes. The pyrazole sulphonamide NMT inhibitor DDD85646 is 13–23-fold less potent against recombinant *Tc*NMT than *Tb*NMT (*T. brucei* NMT), with *K*_i_ values of 12.7 and 22.8 nM respectively, by scintillation proximity or coupled assay methods. DDD85646 also inhibits growth of *T. cruzi* epimastigotes (EC_50_=6.9 μM), but is ~1000-fold less potent than that reported for *T. brucei*. On-target activity is demonstrated by shifts in cell potency in lines that over- and under-express NMT and by inhibition of intracellular N-myristoylation of several proteins in a dose-dependent manner. Collectively, our findings suggest that N-myristoylation is an essential and druggable target in *T. cruzi.*

## Abstract 2

The present study shows that *N*-myristoyltransferase is essential for growth of *Trypanosoma cruzi*, the parasite responsible for Chagas’ disease. The kinetic properties of the enzyme are described along with evidence that growth is specifically inhibited by blocking N-myristoylation in the parasite.

## INTRODUCTION

The protozoan parasite *Trypanosoma cruzi* is the causative agent of Chagas’ disease, which is endemic in Latin American countries. There are an estimated 8–10 million infected individuals worldwide, with an annual death toll of ~10000 per annum [1–3]. Migration from endemic countries has also led to the worldwide distribution of Chagas’ disease [1]. The acute stage of this disease often has very mild and non-specific symptoms that occur 4–8 weeks post-infection, resulting in only 1–2% of all infected individuals being diagnosed in this stage [4]. Approximately 30% of infected individuals go on to develop the chronic disease, most often characterized by heart abnormalities, and to a lesser extent, mega-organ disease affecting the digestive tract [2]. To date, benznidazole and nifurtimox are the only approved drugs available for the treatment of Chagas’ disease. Prolonged treatment with these nitroimidazoles during the acute stage cures up to 70% of individuals; however, the efficacy of these drugs significantly decreases in the chronic stage [5]. Both therapies have been associated with severe toxic side effects that can lead to the interruption or discontinuation of treatment in as many as 30% of cases [6,7]. At present, there are two drugs being clinically assessed for the treatment of asymptomatic chronic Chagas’ disease, posaconazole (Merck; ClinicalTrials.gov Identifiers NCT01377480 and NCT01162967) and E1224 (Eisai; ClinicalTrials.gov Identifier NCT01489228). However, bearing in mind the high levels of drug candidate attrition in the clinical trials process, there remains an urgent need to identify new drug targets and better drugs to treat this disease.

The enzyme NMT (*N-*myristoyltransferase; EC 2.3.1.97) catalyses the co- and post-translational addition of myristic acid (C_14:0_) on to the N-terminal glycine residue of specific proteins [8,9]. This irreversible modification plays an important role in the correct cellular localization and biological function of the modified proteins. This enzyme has been extensively studied in a number of organisms including the trypanosomatid parasites *Trypanosoma brucei* and *Leishmania major* [10–15]. In these parasitic organisms, NMT has been demonstrated to be essential for viability either by classical gene knockout with episomal rescue or by RNAi, indicating that the N-myristoylation of certain proteins is a key biological process. Moreover, in the African trypanosome, NMT is now pharmacologically validated with compounds such as DDD85646 that specifically inhibit the enzyme and are curative in the mouse model of stage one African sleeping sickness [13]. Amino acid sequence comparisons indicate that the *T. cruzi* enzyme is approximately 60% identical to those of *Leishmania* spp. and various African trypanosomes. Although metabolic labelling studies in the parasite have confirmed that multiple proteins are N-myristoylated [16], *T. cruzi* NMT has not been characterized biochemically or assessed for essentiality or druggability. With this in mind, in our present study, we utilize both genetic and chemical approaches to assess the essentiality of the enzyme in *T. cruzi*.

## MATERIALS AND METHODS

### Parasite and mammalian cell culture

*T. cruzi* epimastigotes from the Silvio strain (MHOM/BR/78/Silvio; clone X10/7) were grown at 28°C in RTH/FBS [RPMI 1640 medium supplemented with trypticase, haemin, Hepes and 10% heat-inactivated FBS (PAA Laboratories; now GE Healthcare)] [17]. {The Silvio strain, originally isolated from a 19-year-old male patient (Silvio B.S.) living in Pará, Brazil [18], is also incorrectly referred to as the Sylvio strain in the literature.} Clone Silvio X10/7A, used in subsequent experiments, was generated by limiting dilution. Stationary-phase epimastigote cultures containing metacyclic trypomastigotes were used to infect Vero cells. Trypomastigotes were recovered from Vero cell monolayers infected with Silvio X10/7A at 5–6 days post-infection [19]. For infectivity studies, Vero cells were infected with transgenic *T. cruzi* trypomastigotes using a multiplicity of infection of 5:1. Free-swimming trypomastigotes were washed off after 12 h and the infected cells were re-plated into 384-well plates (Corning® CellBIND®). After 72 h the cells were fixed in PBS containing 1% formaldehyde before staining with 5 μg·ml^−1^ Hoechst 33342 in PBS containing 0.01% Triton X-100. Plates were imaged using a high content microscope (Operetta, PerkinElmer), and the images captured were processed using an automated image analysis software (Columbus, PerkinElmer) to determine the percentage of infected cells and the mean number of parasites per infected Vero cell. Vero cells (*Cercopithecus aethiops* kidney cells; ATCC® CCL-81™) were cultured in DMEM (Dulbecco's modified Eagle's medium; Lonza) supplemented with 10% heat-inactivated FBS at 37°C with 5% CO_2_ [20].

### Cloning, expression and purification of recombinant *Tc*NMT (*T. cruzi* NMT)

The *NMT* ORF was identified from the Silvio X10/1 genome by BLAST, using the CL-Brenner sequence (TriTrypDB accession number TcCLB.511283.90) as a search template [21]. Primers designed against this sequence, *Tc*NMT-pET15b-TEV_s and *Tc*NMT-pET15b-TEV_as (Table 1), were used to amplify the *NMT* ORF from Silvio X10/7A genomic DNA using Pfu DNA polymerase (Promega). The resulting PCR product was cloned into Zero Blunt® TOPO® and sequenced. *TcNMT* was excised from Zero Blunt® TOPO®-*Tc*NMT by digestion with the appropriate restriction enzymes and ligated directly into linearized pET15b-TEV.

**Table 1:** Primers used in the present study Complementary sequences to ORFs are capitalized. Restriction sites are underlined.

Primer	Sequence
*Tc*NMT-pTREX_s	5′-gaattcATGGCAGAAGAGGGTTCAGGTTTACATCAG-3′
*Tc*NMT-pTREX_as	5′-ctcgagCTATAGCATGAACAATCCCACGTCACTTGG-3′
*Tc*NMT-pET15b-TEV_s	5′-catATGGCAGAAGAGGGTTCAGGTTTACATCAG-3′
*Tc*NMT-pET15b-TEV_as	5′-ggatccCTATAGCATGAACAATCCCACGTCACTTGG-3′
5′-UTR-NotI _s	5′-ataagaatgcggccgcGTGATCTTCTCAACAACAAAAATGGATGA-3′
5′-UTRHindIII/PmeI_as	5′-gtttaaacttacggaccgtcaagcttTCCTTCAAAAGGCGATCAAGTCCA- AAATTAC-3′
3′-UTR-PmeI/BamHI_s	5′-gacggtccgtaagtttaaacggatccGATGCGGGCGGAATTTAGGAGAGA- AGT-3′
3′-UTR-NotI_as	5′-ataagtaagcggccgcCCGCATCCAGCAGATGGATTAATCACCGT-3′

The resulting pET15b-*Tc*NMT expression construct was transformed into Rosetta™ (DE3)pLysS competent cells and recombinant expression was carried out in auto-induction media [22] at 20°C for 48 h with agitation at 200 rev./min. The cells were harvested (20 min, 4°C and 5020 ***g***), resuspended in lysis buffer {25 mM Tris, 500 mM NaCl, 25 mM imidazole, 1 mM TCEP [tris-(2-carboxyethyl)phosphine]/HCl, pH 8.5, DNAse I (Sigma) and cOmplete EDTA-free protease inhibitors (Roche)} and lysed at 30000 psi (1 psi=6.9 kPa) using a Constant Systems cell disruptor. Soluble protein was recovered by centrifugation (30 min, 4°C and 40000 ***g***) and filtered (0.2 μm Sartorius) before loading on to a pre-equilibrated HisTrap HP 5 ml column (GE Healthcare). The protein was eluted using a gradient of 25–500 mM imidazole. Fractions containing NMT were identified by SDS/PAGE (4–12% gel), pooled and dialysed into buffer A (25 mM Tris, 25 mM NaCl and 1 mM TCEP, pH 8.5). The dialysed protein was loaded on to a 5 ml HiTrap Q HP column (GE Healthcare) and eluted with a gradient of NaCl (25–500 mM) in buffer A. Pooled fractions containing NMT were further purified by size exclusion on a Superdex 75 26/60 column equilibrated in buffer B (25 mM Tris/HCl, 150 mM NaCl and 1 mM TCEP, pH 8.5). The purity and mass of the recovered recombinant NMT was assessed by SDS/PAGE and MALDI–TOF carried out by the FingerPrints Proteomics service at the University of Dundee. The oligomeric structure was characterized by size-exclusion chromatography using a Superdex 200 300/10 GL column (GE Healthcare) equilibrated with buffer B.

## GENERATION OF KNOCKOUT, OVEREXPRESSION AND RECOVERY CONSTRUCTS

The primers used to generate constructs for genetic manipulation were designed using the *Tc*NMT X10/1 and flanking sequences in TriTrypDB as a template (Table 1). The accuracy of all assembled constructs was verified by sequencing. NMT gene replacement cassettes were generated by amplifying a region of DNA encompassing 449 bp of the 5′-UTR, the ORF and 449 bp of the 3′-UTR of *Tc*NMT from genomic DNA with primers 5′-UTR-NotI_s and 3′-UTR-NotI_as, using Pfu DNA polymerase. This sequence was then used as a template for the amplification of the individual regions used in the assembly of replacement cassettes containing the selectable drug resistance genes *PAC* (puromycin *N*-acetyltransferase) and *HYG* (hygromycin phosphotransferase), exactly as described previously [23]. To generate a construct for use as both a recovery and NMT-overexpressing vector in knockout and WT (wild-type) parasites, NMT was amplified from genomic DNA using the primers *Tc*NMT-pTREX_s and *Tc*NMT-pTREX_as and cloned into the constitutive expression vector pTREX [24] using the EcoRI and XhoI cloning sites.

### Generation of transgenic *T. cruzi* cell lines

Transfections of *T. cruzi* epimastigotes were carried out using an Amaxa Nucleofector™ electroporator, as described previously [25]. A total of 5–10 μg of DNA was transfected into early- to mid-log epimastigotes (1×10^7^), suspended in Human T-cell Nucleofector™ solution (100 μl; Lonza), using the program U-33. At 24 h following transfection, 10 μg·ml^−1^ puromycin (Sigma), 250 μg·ml^−1^ G418 (Gibco®) or 500 μg·ml^−1^ hygromycin (Roche) was added to cultures of transgenic parasites. Following drug selection, the parasites were cloned on to semi-solid agar plates [1% Agar Noble (Difco™) and RTH/FBS] containing 20 μg·ml^−1^ puromycin, 500 μg·ml^−1^ G418 or 750 μg·ml^−1^ hygromycin, as appropriate. After 2–3 weeks at 28°C, colonies were picked and grown in fresh RTH/FBS plus the appropriate drug.

### *In vitro* drug sensitivity assays

To examine the effects of test compounds on growth, triplicate epimastigote cultures were seeded with 1×10^5^ cells·ml^−1^. Parasites were grown in 10-ml cultures in the presence of drug for 120 h. Cells were fixed in PBS (137 mM NaCl, 2.7 mM KCl, 10 mM Na_2_HPO_4_ and 1.8 mM KH_2_PO_4_) containing 1% formaldehyde and manually counted using a Neubauer haemocytometer. Data were processed using GraFit (version 5.0.4; Erithacus software) and fitted to a 2-parameter equation (eqn 1) to obtain the effective EC_50_:

(1) $$y=\frac{100}{1+{\left(\frac{\left[I\right]}{{\mathrm{EC}}_{50}}\right)}^{m}}$$

In this equation, [I] represents inhibitor concentration and *m* is the slope factor. The data are presented as the means±S.D.

### Quantification of cellular levels of NMT in lysates

Epimastigotes and trypomastigotes were harvested by centrifugation (15 min, 20°C, 1620 and 2000 ***g*** respectively) and washed twice in PBS. Amastigotes were purified from a mixed population of trypomastigotes and amastigotes released from an infected Vero cell monolayer [25a]. Briefly, parasites were collected by centrifugation (10 min, 20°C, 4000 ***g***) and the pellet incubated for 3 h at 37°C overlaid with DMEM/FBS. Motile trypomastigotes released into the supernatant were removed and the pellet was resuspended in DMEM/FBS. This process was repeated twice to produce a pure population of amastigotes (~95%). Cells (5×10^7^) were resuspended in Laemmli buffer (Bio-Rad Laboratories) and heated at 95°C for 10 min. The equivalent of 1×10^7^ cells were separated by SDS/PAGE on a 4–12% NuPAGE® gel. Cellular proteins were transferred on to Protran™ nitrocellulose membrane (Whatman) by electrotransfer. Membranes were probed with primary rat antisera generated against either *Tc*NMT or *Tc*TryR (*T. cruzi* trypanothione reductase) [26] (both 1:500 dilution) before probing with an HRP (horseradish peroxidase)-conjugated rabbit anti-rat polyclonal secondary serum (1:10000; Dako). *Tc*NMT-specific polyclonal antiserum was raised against the recombinant His_6_–*Tc*NMT (CL-Brenner) in adult male Wistar rats, as described previously [27]. Immunization protocols were approved by the University Welfare and Ethical Use of Animals Committee and were performed under the Animals (Scientific Procedures) Act 1986 in accordance with the European Communites Council Directive (86/609/EEC). The blot was developed using ECL detection reagent kit (GE Healthcare) and exposed to Amersham Hyperfilm™ ECL (GE Healthcare). The developed film was scanned and the protein bands were quantified by densitometry with ImageJ (NIH).

### Southern blot analyses of transgenic *T. cruzi* cell lines

The ORFs of *TcNMT*, *PAC* and *HYG* were amplified by PCR (using the primers described previously for the cloning of *TcNMT* and knockout constructs) using the PCR DIG Probe Synthesis Kit (Roche). The resulting DIG (digoxigenin)-labelled products were used as probes. Samples of genomic DNA (5 μg) from WT and transgenic cell lines were digested with appropriate restriction endonucleases, the digestion products were then separated on a 0.8% agarose gel and transferred to a positively charged nylon membrane (Roche). The membrane was hybridized overnight in DIG Easy Hyb solution (Roche) at 42°C with the DIG-labelled probes (2 μl of PCR product). Following hybridization, membranes were washed twice in low stringency conditions (25°C, 5 min, 2× SSC buffer with 0.1% SDS) and twice in high stringency conditions (68°C, 15 min, 0.5× SSC with 0.1% SDS), where 1× SSC comprises 150 mM NaCl and 50 mM sodium acetate, pH 7.0. The bound probe was detected using the DIG immunological detection kit (Roche) as per the manufacturer's instructions.

### Detection of cellular N-myristoylation

Mid-log epimastigotes were harvested by centrifugation and resuspended at 1×10^7^ cells·ml^−1^ in fresh RTH/FBS. Various concentrations of DDD85646 (0, 12.5, 25, 50 and 100 μM) were added to epimastigote cultures 30 min before the addition of 50 μM Click-IT® myristic acid (Invitrogen) and cultures were then incubated for a further 5 h. Following incubation, cells were washed (three times in PBS), the resulting cell pellet was resuspended in lysis buffer (150 μl, 50 mM Tris/HCl, pH 7.4, 150 mM NaCl, 1% sodium deoxycholate, 0.1% SDS, 1% Triton X-100 and a cOmplete mini EDTA-free protease inhibitor cocktail tablet) and incubated on ice for 1 h. Lysates were clarified by centrifugation (10 min, 4°C and 14000 ***g***) and quantified with the Bio-Rad Laboratories protein assay using BSA as a standard. IRDye® 800CW alkyne (LI-COR Biosciences) was ligated to Click-IT® myristic acid using the Click-IT® protein reaction buffer set (Invitrogen) and methanol/chloroform precipitated, according to the manufacturer's instructions. Treated lysates (12 μg) were separated by SDS/PAGE, fixed in 10% acetic acid and 40% methanol. The fixed gel was washed in 0.2 M NaOH for 1 h before washing briefly in H_2_O and imaged by in-gel fluorescence using an Odyssey Sa infrared imaging system (LI-COR Biosciences). Quantification of band intensities was carried out using Image Studio Lite (version 3.1; LI-COR Biosciences). Cells not labelled with azidomyristate were used for a background fluorescence measurement to correct the values obtained for N-myristoylated proteins. Intensities are expressed as a percentage of the no drug control.

### Metabolic labelling

Parasites were incubated in a methionine-free RTH/FBS medium that was supplemented with 10 μCi·ml^−1^ L-[^35^S]methionine (PerkinElmer). After incubating with the same concentrations of inhibitor and azidomyristate as mentioned above, the parasites were washed three times in PBS and boiled in Laemmli buffer for 10 min. A total of 5×10^6^ parasites per lane were separated by SDS/PAGE and stained with Coomassie Blue. The gel was incubated in EN^3^HANCE™ solution (PerkinElmer) as per the manufacturer's protocol and then gel dried. The gel was exposed to BioMax MS film (Kodak) using a BioMax TranScreen LE (Kodak) for 8 h.

### Kinetic analysis of *Tc*NMT

Kinetic analysis [*K*_m(app)_ and *k*_cat_ values] of *Tc*NMT activity was performed at 30°C using a previously published coupled-enzyme spectrophotometric assay monitoring the increase in absorbance at 340 nm [28]. Each 0.25 ml assay contained 50 mM Tris, 0.5 mM EDTA, 0.5 mM EGTA, 1.25 mM DTT, 0.1% Triton X-100, 40 mM pyruvic acid, 0.125 units·ml^−1^ pyruvate dehydrogenase, 0.2 mM thiamine pyrophosphate, 40 μM myristoyl-CoA and 2.5 mM NAD^+^, adjusted to pH 7.4 with HCl. *K*_m(app)_ values were determined for a biotinylated peptide substrate derived from amino acids 2–15 of *T. brucei* [13] and *T. cruzi* CAP5.5 (cytoskeleton-associated protein 5.5) (*Tc*CAP5.5 GCCASKEKQPRPGAK[biotin], *Tb*CAP5.5 GCGGSKVKPQPPQAK[biotin], custom synthesized by Pepceuticals) and for myristoyl-CoA (Sigma). The IC_50_ value of DDD85646 for recombinant NMT was determined using this coupled-enzyme assay. The *K*_i(app)_ was determined by fitting the resulting data to the Morrison equation (eqn 2), allowing the true *K*_i_ value to be determined using eqn (3). In a comparative study, the kinetic parameters of *Tc*NMT (5 nM per assay) were also determined using a scintillation proximity method, as described previously [13,29]. The myristoyl-CoA *K*_m(app)_ was determined using CAP5.5 at 600 μM or 50 μM in the coupled-enzyme and scintillation proximity assays respectively. The CAP5.5 *K*_m(app)_ values were determined using either 40 μM or 125 nM in the coupled-enzyme or scintillation proximity assays.

(2) $$\begin{array}{ccc} & & \frac{v_{i}}{v_{0}}=\\ & & \frac{\left({\left[E\right]}_{T}-{\left[I\right]}_{T}-K_{i\left(\mathrm{app}\right)}\right)+{\sqrt{{\left({\left[E\right]}_{T}-{\left[I\right]}_{T}-K_{i\left(\mathrm{app}\right)}\right)}^{2}+4{\left[E\right]}_{T}\left[I\right]}}_{T}}{2{\left[E\right]}_{T}} \end{array}$$

(3) $$K_{i}=\frac{K_{i(\mathrm{app})}}{\left(1+\frac{\left[S\right]}{K_{m}}\right)}$$

## RESULTS

### Generation of an NMT ‘rescued’ DKO (double knockout) cell line

**Figure 1:**
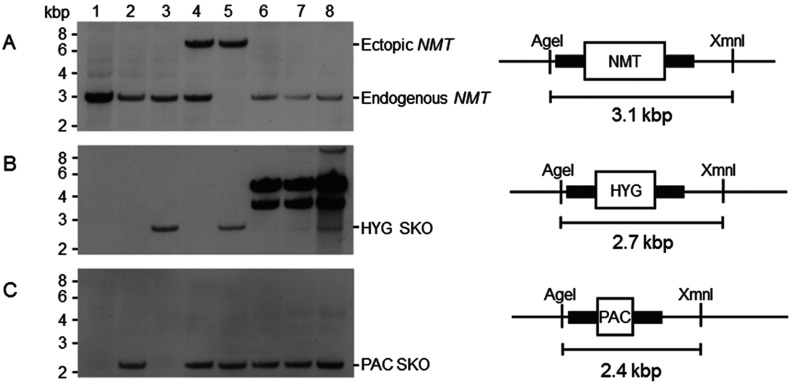
Genotypic analysis of WT, SKO and rescue DKO cell lines Southern blot analysis of AgeI and XmnI digested genomic DNA (~5 μg) from WT *T. cruzi* (clone Silvio X10/7A) cells (lane 1), *NMT* SKO (*PAC*) cells (lane 2), *NMT* SKO (*HYG*) cells (lane 3), *NMT* SKO (*PAC*) cells constitutively expressing NMT (lane 4), *NMT* DKO (*PAC* and *HYG*) cells constitutively expressing NMT (lane 5) and ‘pseudo’ *NMT* DKO (*PAC* and *HYG*) cells (lanes 6–8). The maps show the predicted fragment sizes for the WT and for correct replacement with drug resistance markers. Southern blots were probed with (**A**) *NMT* ORF, (**B**) *HYG* and (**C**) *PAC*.

Restriction enzyme digestion and Southern blotting analysis of *T. cruzi* X10/7A DNA indicated that *NMT* is a single copy gene per haploid genome (results not shown). DNA sequencing of PCR products gave identical amino acid sequences apart from a serine or proline residue at position 150, probably due to allelic variation. The essentiality of NMT in *T. cruzi* epimastigotes was then assessed using a classical two-step gene replacement strategy where *NMT* is sequentially replaced by homologous recombination with drug resistance genes and drug selection (Figure 1). The first gene copy of *NMT* could be successfully replaced with either hygromycin (*HYG*) or puromycin (*PAC*) resistance genes resulting in two independent SKO (single knockout) cell lines (Figures 1B, lane 3, and 1C, lane 2). Loss of a single allelic copy of *NMT* did not markedly alter the growth rate of SKO parasites. Several attempts were made to directly replace the remaining allelic copy of *NMT* in the SKO-*PAC* clone with *HYG*. In two out of three attempts, epimastigotes that were resistant to both hygromycin and puromycin were recovered following transfection. On the remaining occasion, no live parasites were recovered. Southern blot analysis of genomic DNA isolated from clones of putative DKO parasites revealed that in all cases an endogenous copy of *NMT* was retained (Figure 1A, lanes 6–8) along with a copy of *PAC* at the *NMT* locus (Figure 1C, lanes 6–8). Moreover, probing these blots with the *HYG* probe showed that this drug resistance gene had not integrated into the *T. cruzi* genome (Figure 1B, lanes 6 and 7). PCR of these failed DKO attempts suggest that the *HYG* resistance gene is present as a multicopy episome. In another of these clones, *HYG* was not only present as an episomal copy, but also integrated at the *NMT* locus with retention of a copy of *NMT* (Figures 1A and 1B, lane 8). We have not investigated whether the latter is due to amplification of all or part of the *NMT* chromosome resulting in aneuploidy, as has been observed in *Leishmania* spp. [30].

Owing to the failure to directly produce *NMT* DKO epimastigotes, a ‘rescued’ DKO cell line was generated. First, a constitutively expressed ectopic copy of *NMT* was targeted to the ribosomal locus of SKO-*PAC* parasites (Figure 1A, lane 4). Only then was it possible to replace the last allelic copy of *NMT* in cells, due to the presence of an episomal copy of the gene (Figure 1A, lane 5). These findings provide strong evidence that NMT is essential for growth and survival of *T. cruzi* epimastigotes *in vitro*.

### Infectivity of transgenic parasites

**Figure 2:**
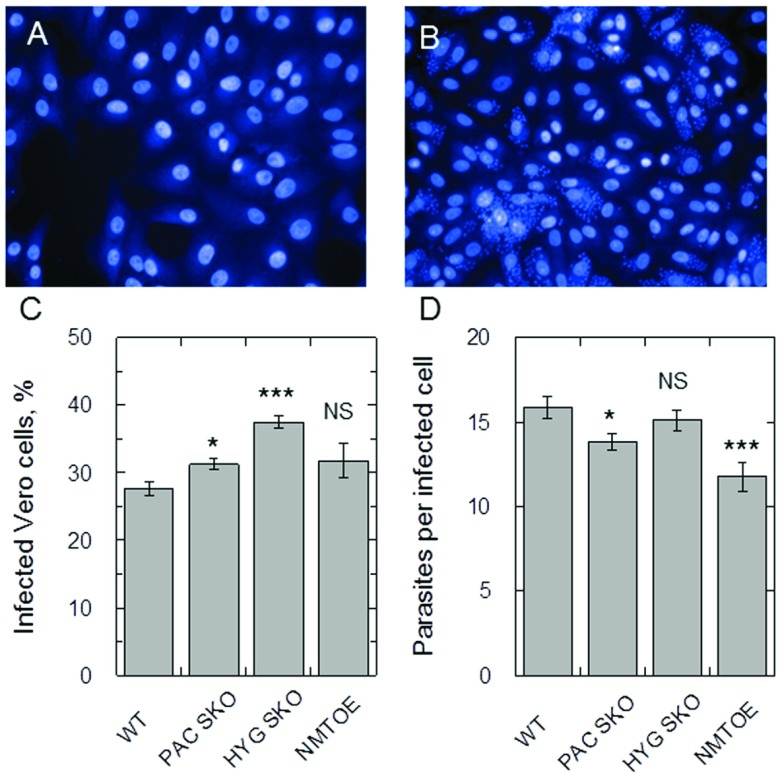
Infectivity of transgenic *T. cruzi* parasites (**A**) Uninfected Vero cells stained with Hoechst 33342. (**B**) Vero cells infected with WT *T. cruzi*. (**C**) The percentage of Vero cells infected with WT, SKO-*PAC*, SKO-*HYG* and NMT^OE^ transgenic parasites. Differences in the percentage of WT compared with SKO-*PAC* and SKO-*HYG* parasites were confirmed to be statistically significant (*P<0.01) using an unpaired Student's *t* test. (**D**) The mean number of amastigotes per infected Vero cell of WT, SKO-*PAC*, SKO-*HYG* or NMT^OE^ parasites. Differences in the percentage of WT compared with SKO-*PAC* and NMT^OE^ parasites were confirmed to be statistically significant (*P*<0.01) using an unpaired Student's *t* test (*P<0.05, ***P<0.001). A total of 24 measurements were made for each parameter. Data are shown as means±S.E.M. NS, not significant.

The ability to infect Vero cells was quantified to determine whether the presence of an ectopic copy or the deletion of a single allele of *TcNMT* affected the virulence of these parasites. Representative images of uninfected and infected Vero cells are shown (Figures 2A and 2B respectively). The deletion of a single allele in both cases led to a very minor increase in the percentage of infected cells compared with the WT, whereas the presence of an ectopic copy [NMT^OE^ (NMT overexpressor)] had no effect (Figure 2C). Absolute numbers of parasites per infected Vero cell were also monitored (Figure 2D). Vero cells infected with SKO-*PAC* and NMT^OE^ parasites were found to have marginally reduced parasite loads compared with WT. Despite the statistical differences between some, but not all, cell lines, these changes are not relevant biologically as all lines showed similar infection profiles.

### Expression of NMT in *T. cruzi* life-cycle stages

**Figure 3:**
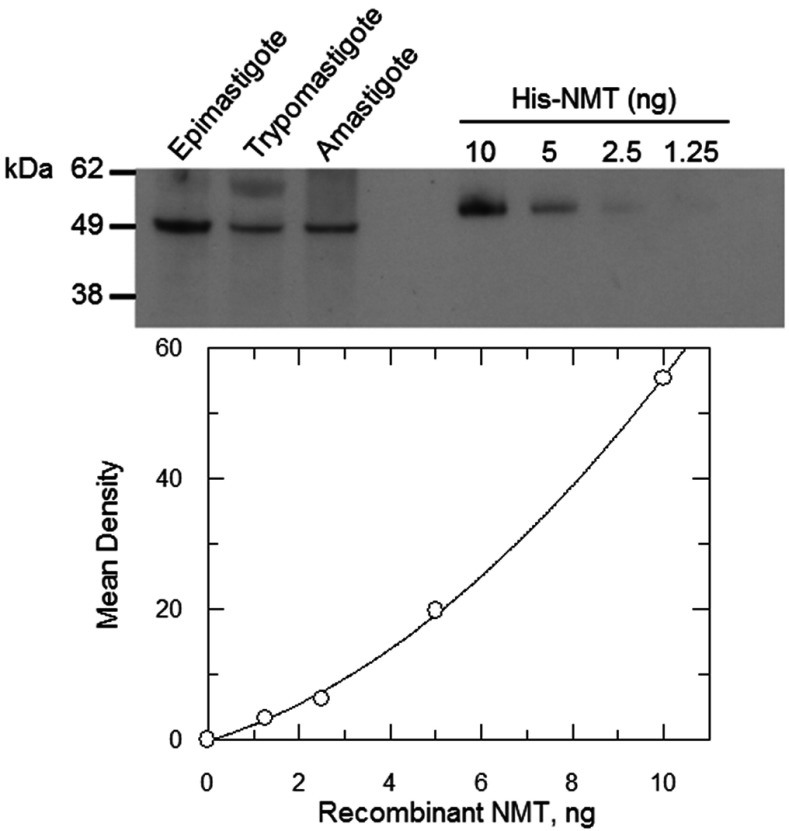
Cellular levels of NMT in *T. cruzi* life-cycle stages Immunoblots of whole cell extracts (equivalent of 1×10^7^ parasites in each lane) from *T. cruzi* epimastigotes, trypomastigotes and amastigotes were probed with *Tc*NMT-specific polyclonal antiserum. Known amounts of purified recombinant *Tc*NMT were loaded as standards for the quantification of the cellular levels of NMT. The difference in size between recombinant and cellular NMT is due to the His_6_-tag on the recombinant protein.

For technical reasons, it is not possible to genetically validate *NMT* in the clinically relevant non-dividing trypomastigote stage and intracellular amastigote stage by gene knockout. However, we were able to confirm that NMT is expressed in all stages of the parasite's life cycle by probing an immunoblot of crude lysates with a *Tc*NMT-specific antiserum (Figure 3). Single bands of approximately 53 kDa, close to the predicted molecular mass of NMT (51.4 kDa), were detected in all three lysates indicating that NMT is expressed at all stages of the parasite life cycle. The cellular concentration of NMT in each of these parasite stages was determined by densitometry and previously published cell volumes [31]. Using this information, NMT concentrations in each stage of the parasite were estimated to be within a 2-fold range; 1.2, 2.1 and 2.5 μM in the epimastigote, trypomastigote and amastigote respectively.

### Sensitivity to DDD85646 shifts with NMT expression levels

**Figure 4:**
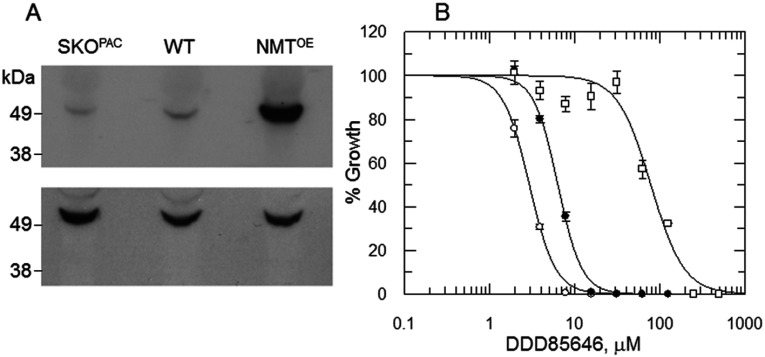
Effects of NMT modulation on DDD85646 susceptibility (**A**) Immunoblots of whole cell extracts (equivalent of 1×10^7^ parasites in each lane) of WT, NMT SKO and NMT-overexpressing epimastigotes were probed with *Tc*NMT-specific polyclonal antiserum. A duplicate blot was probed with antiserum against *Tc*TryR to act as a loading control. (**B**) EC_50_ values were determined for DDD85646 against WT (closed circles), SKO (PAC) (open circles) and NMT-overexpressing parasites (open squares). EC_50_ values of 6.3±0.1, 2.9±0.04 and 78.6±4.6 μM were determined for DDD85646 against WT, SKO and NMT-overexpressing cell lines respectively. Data are shown as means±S.D. for triplicate cultures.

The pyrazole sulphonamide DDD85646 has been shown to specifically inhibit *Tb*NMT (*T. brucei* NMT) *in vitro* and cure the stage 1 murine model of human African trypanosomiasis [13]. To establish whether this inhibitor can also chemically target the *T. cruzi* enzyme, the comparative sensitivity of WT epimastigotes and transgenic cell lines with different levels of NMT to DDD85646 was determined. In the first instance, altered levels of NMT expression in transgenic parasites were confirmed by Western blot, using *Tc*TryR as a loading control (Figure 4A). Cellular levels of NMT were analysed in WT parasites, the SKO cell line generated previously (SKO-*PAC*) and in an NMT overexpressing cell line (NMT^OE^) which was generated by transfecting pTREX-NMT into WT epimastigotes. Densitometry revealed that SKO-*PAC* parasites contained NMT protein levels ~2.5-fold lower than the WT, with levels in the NMT^OE^ epimastigotes ~7.6-fold higher. Varying the cellular levels of NMT within these parasites was found to markedly alter their sensitivity to DDD85646 with WT, SKO-*PAC* and NMT^OE^ cell lines having EC_50_ values of 6.3, 2.9 and 78.6 μM respectively (Figure 4B). The clear relationship observed between the levels of NMT expression and the sensitivity of the parasites for this compound confirms that *Tc*NMT is specifically targeted by DDD85646 and thus may be druggable in *T. cruzi.* There was no selectivity between the amastigote and Vero cells with DDD85646 [EC_50_ values of ≥8.7±0.8 μM and 6.7±1 μM (*n*=4) respectively]. The actual EC_50_ value for the amastigote may be higher as the parasite cannot replicate in the absence of the host cell.

### DDD85646-mediated inhibition of N-myristoylation

**Figure 5:**
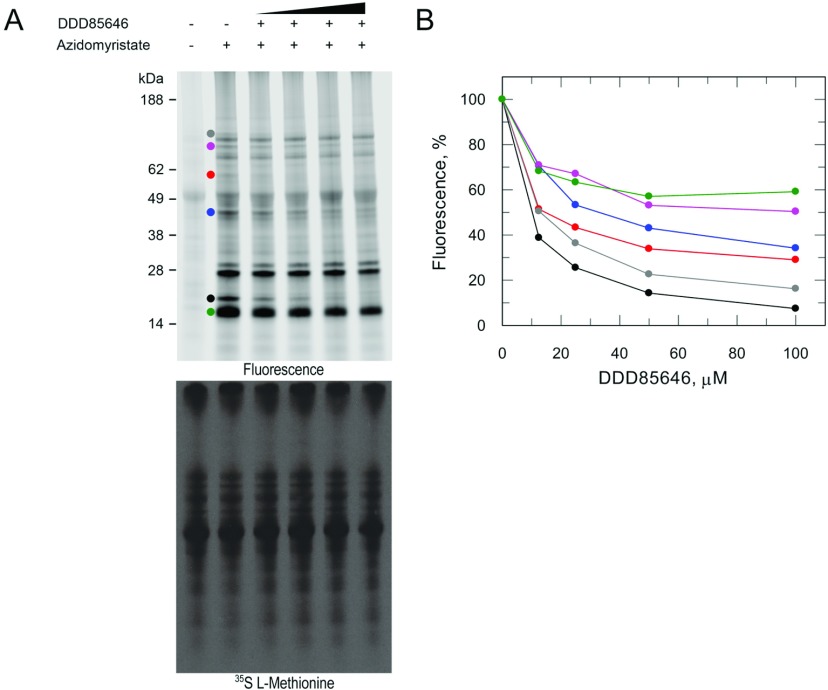
DDD85646-mediated inhibition of cellular N-myristoylation Mid-log epimastigotes were pre-treated with varying concentrations of DDD85646 (0–15×EC_50_) for 5.5 h. (**A**) N-myristoylated proteins were detected by click chemistry ligation of an alkyne fluorescent dye on to azidomyristate-labelled proteins (upper panel) and protein synthesis assessed by L-[^35^S]methionine labelling of parasites (lower panel). Circles highlight bands that are sensitive to NMT inhibition that were quantified in (**B**). (**B**) Reduction in fluorescence intensity as a function of DDD85646 concentration.

To confirm DDD85646-mediated inhibition of N-myristoylation within *T. cruzi* epimastigotes, parasites were pre-treated with a range of inhibitor concentrations for 30 min. N-azidomyristoylated proteins were detected by in-gel fluorescence. It is evident that there is some non-specific interaction of the dye with an unlabelled 49 kDa protein (Figure 4A, upper panel, lane 1). Labelling parasites with this myristic acid analogue led to the NMT-mediated incorporation of azidomyristate into multiple *T. cruzi* proteins (Figure 4A, upper panel, lane 2). In parasites treated with DDD85646, we observe that six bands were depleted in a dose-dependent manner which was confirmed by quantifying the fluorescent intensities of the bands (Figures 5A, upper panel, and 5B). The most prominent effect was observed for a ~20 kDa band, where the N-myristoylation of this protein decreased to 40% of the untreated control, at the lowest inhibitor concentration tested (~2×EC_50_). The remaining bands are insensitive over 5.5 h exposure to DDD85646 at the range of concentrations tested. Labelling parasites with L-[^35^S]methionine revealed no inhibition of nascent protein synthesis (Figure 5A, lower panel), indicating that the observed inhibition of N-myristoylation is due to the direct inhibition of cellular NMT. These data further demonstrate the on-target activity of the inhibitor DDD85646 in *T. cruzi*.

### Kinetic characterization of recombinant *Tc*NMT

**Figure 6:**
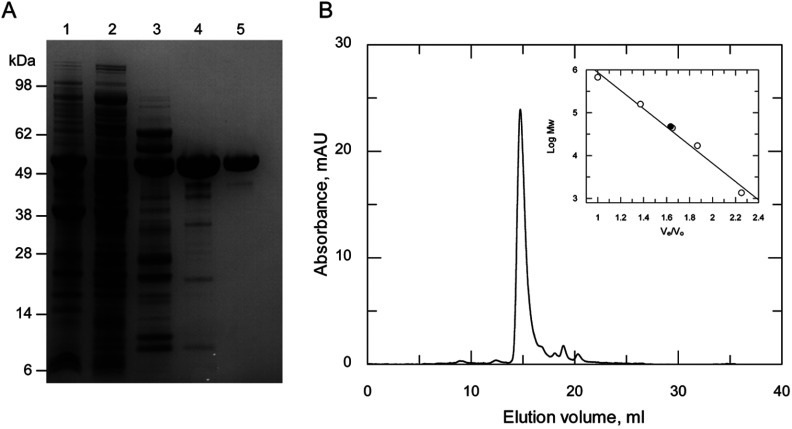
Purification of recombinant *Tc*NMT (**A**) SDS/PAGE of purification of recombinant *Tc*NMT. Lane 1, insoluble fraction of Rosetta™ 2 (DE3)pLysS [pET15b-*Tc*NMT], induced; lane 2, soluble fraction of Rosetta™ 2 (DE3)pLysS [pET15b-*Tc*NMT], induced; lane 3, pooled fractions from Ni^2+^-affinity chromatography; lane 4, pooled fractions from anion exchange chromatography (Q Sepharose); and lane 5, pooled fractions from size-exclusion chromatography. (**B**) Gel filtration profile of the His_6_-tagged *Tc*NMT. The inset shows a plot of *V*_e_/*V*_0_ against the log molecular mass (Mw) of a standard protein mixture (open circles), where *V*_e_ is the elution volume and *V*_0_ is the void volume of the column. The closed circle represents the elution volume of NMT.

In order to facilitate kinetic studies of *Tc*NMT, the recombinant enzyme was expressed and purified to homogeneity. *Escherichia coli* Rosetta™ (DE3)pLysS cells transformed with pET15b-TEV-*Tc*NMT produced soluble and active protein. *Tc*NMT was purified following three chromatographic steps to obtain a yield of 2.5 mg·l^−1^ (Figure 6A). Analysis of the recombinant protein by size-exclusion chromatography revealed that His_6_–NMT elutes primarily as a monomer at ~47.4 kDa, close to the predicted molecular mass of 53.7 kDa (Figure 6B). This was confirmed by MS to be 53.7 kDa for the tagged recombinant protein by MALDI–TOF analysis.

Multiple assays already exist for the kinetic characterization of NMTs using HPLC, ELISA, scintillation proximity assay or spectrophotometric methodologies [9,29,32,33]. In the present study, we have compared the scintillation proximity assay with a modified version of a coupled-enzyme spectrophotometric assay [28]. The basic kinetic parameters of *Tc*NMT [*K*_m(app)_ and *k*_cat_] were measured in these assays for CAP5.5, a protein known to be N-myristoylated in *T. brucei* [34] (Table 2). Synthetic peptides based on the amino acids 2–15 of CAP5.5 from both *T. brucei* and *T. cruzi* were used as substrates in these assays. In the coupled-enzyme assay, the *K*_m_ value determined for *Tb*CAP5.5 was ~21-fold higher than observed for *Tc*CAP5.5, but the catalytic efficiencies (*k*_cat_/*K*_m_) of both substrates were found to be similar. For reasons of cost, it was not possible to determine the *K*_m_ values in the presence of saturating concentrations of myristoyl-CoA using the scintillation proximity assay, allowing only a *K*_m(app)_ value to be determined for each peptide. Using this assay, the *K*_m(app)_ values for the peptide substrates were very similar at 1.6 and 2.2 μM. In the coupled-enzyme assay (in the presence of 600 μM *Tb*CAP5.5), the *K*_m(app)_ value of the myristoyl-CoA substrate was 6.2±0.6 μM, which is not statistically different from the value of 5.3±1.0 μM determined in the scintillation proximity assay (in the presence of 50 μM *Tc*CAP5.5) (*P* =  0.252) Student's *t* test.

**Table 2:** Kinetic characterization of recombinant *Tc*NMT Data for the *Tb*NMT column are taken from [13,29]. n.d., not determined.

Parameter	Coupled-enzyme assay	Scintillation proximity assay	*Tb*NMT
*K*_m(app)_ (μM)			
Myristoyl-CoA	6.2±0.6	5.3±1.0	1.78
*Tb*CAP5.5	250±28	2.2±0.2	11.3
*Tc*CAP5.5	12.1±1	1.6±0.15	–
*k*_cat_ (s^−1^)			
Myristoyl-CoA	0.34±0.01	n.d.	–
*Tb*CAP5.5	2.11±0.05	n.d.	–
*Tc*CAP5.5	0.15±0.003	n.d.	–
*k*_cat_/*K*_m_ (M^−1^·s^−1^)			
Myristoyl-CoA	54.8×10^3^	–	–
*Tb*CAP5.5	8.44×10^3^	–	–
*Tc*CAP5.5	12.4×10^3^	–	–
Inhibition by DDD85646			
*K*_i(app)_ (nM)	41.2±5.4*	16.6±4.4†	1.44
*K*_i_ (nM)	22.8	12.7	1.04

*Determined using 150 nM *Tc*NMT, 200 μM *Tb*CAP5.5 and 40 μM myristoyl-CoA.

†Determined using 5 nM *Tc*NMT. 0.5 μM *Tc*CAP5.5 and 0.125 μM myristoyl-CoA.

### Inhibition of recombinant *Tc*NMT by DDD85646

DDD85646 is a potent inhibitor of *T. brucei* recombinant NMT [*K*_i(app)_=1.44 nM] and inhibits the growth of *T. brucei* bloodstream parasites *in vitro* at similar concentrations (EC_50_=2.1 nM) [13]. In comparison, we noted that DDD85646 was far less potent against *T. cruzi* epimastigotes (EC_50_=6.3 μM) (Figure 4B). Since we have demonstrated that DDD85646 specifically inhibits *Tc*NMT *in vitro*, the drop-off in cellular potency could be in part explained by differences in active site architecture leading to a decreased affinity for the inhibitor. To test this hypothesis, the *K*_i_ value of DDD85646 was determined against the *T. cruzi* recombinant enzyme using both the scintillation proximity assay and coupled assay (Table 2). Under both sets of assay conditions, the *K*_i_ of DDD85646 was calculated to be ~12.7–22.8 nM, which is 13–23-fold less potent than against the *T. brucei* enzyme. In contrast with *T. brucei*, there is a drop-off in culture potency of two orders of magnitude between target and cell activity.

## DISCUSSION

The paucity of validated drug targets in *T. cruzi* has severely hampered the search for better and more effective treatments for Chagas’ disease. Previous studies have shown that the enzyme encoded by the *NMT* gene is essential for the survival of many eukaryotic organisms [10,12,35,36], including the related trypanosomatids *L. major* and *T. brucei* [12]. Metabolic labelling studies in *T. cruzi* have already revealed that N-myristoylation occurs in this parasite and plays a role in the correct cellular localization of the flagellar calcium-binding protein [16,37]. The genetic studies investigated in the present study indicate that *TcNMT* is an essential gene in the epimastigote stage of the parasite, since we were unable to directly replace both endogenous copies of *NMT*, except in the presence of an ectopic copy of the gene. Although we have carried out genetic validation of *Tc*NMT in the epimastigote stage of the parasite, there is clear evidence to show that the enzyme is also present in the clinically relevant stages. Therefore it is likely that N-myristoylation is also an essential cellular process during the trypomastigote and amastigote stages of development.

The comparative profiling of NMT substrate specificities from multiple organisms has revealed that there are subtle species-specific differences in the N-myristoylation motif of protein substrates recognized by each homologue. These differences have already been exploited to generate inhibitors which are up to 560-fold more potent against a fungal enzyme than the human enzyme [38]. Several high-throughput inhibitor-screening programmes have been carried out in recent years with the aim of identifying both potent and selective inhibitors of NMT from the target species [13,39,40]. One such campaign led to the development of DDD85646, a highly potent inhibitor of *T. brucei* and human NMT [13]. Despite selectivity at the target level being only 2-fold, this increases to 200-fold at the cellular level. The reason for biological selectivity is not fully understood and may involve pleiotropic biological effects. Depletion of NMT by RNAi in this parasite leads to impairment of the endocytic pathway [41], a process that is known to involve the N-myristoylated protein *Tb*ARF1 (*T. brucei* ARF1) [42]. Endocytosis and exocytosis in *T. brucei* occurs exclusively from a specialized invagination of the plasma membrane known as the flagellar pocket. Owing to the high endocytic/exocytic rate, the entire plasma membrane of the parasite is turned over in approximately 12 min, considerably faster than that of mammalian macrophages or fibroblasts [43]. Treatment of *T. brucei* with DDD85646 causes a massively enlarged flagellar pocket or ‘big eye’ phenotype [13], as found by RNAi knockdown of either clathrin heavy chain [44] or ARF1 [42], suggesting that endocytosis, but not exocytosis, is inhibited. Curiously, knockdown of NMT itself does not produce this phenotype, despite inhibiting endocytosis [41]. Nonetheless, the marked sensitivity of the *T. brucei* bloodstream parasite to NMT inhibition can be attributed at least partly to the high rate of endocytosis/exocytosis and the consequent high turnover of plasma membrane in the flagellar pocket [13].

Although DDD85646 is a potent inhibitor of the *T. cruzi* enzyme, there is a considerable drop off in potency against the intact parasite (epimastigote or amastigote), in marked contrast with *T. brucei* where DDD85646 is equipotent against both the enzyme and the parasite [13]. The reason for this is not clear, but could be due to differences in the rate of plasma membrane turnover, differences in other essential biological functions requiring N-myristoylation or due to differences in cellular pharmacokinetics of drug uptake or efflux. The kinetics of endocytosis has not been studied in *T. cruzi*. However, it is worth noting that endocytosis in *T. cruzi* epimastigotes occurs principally via another membrane invagination adjacent to the flagellar pocket (the cytostome) and not the flagellar pocket itself [45].

Our studies clearly demonstrate that NMT is an essential and druggable enzyme in *T. cruzi*, thus it is entirely plausible that parasite-specific N-myristoylated proteins may also be potential drug targets in their own right. To date, only two *T. cruzi* proteins (flagellar calcium-binding protein and phosphoinositide-specific phospholipase C) have been definitively confirmed to be N-myristoylated [16,37], although two studies have predicted many proteins may undergo this modification [46,47]. Although our studies identify at least ten distinct bands, treatment of epimastigotes with DDD85646 was only able to specifically block the N-myristoylation of six *in vitro* under the experimental conditions used in the present study. Although it is possible to theoretically predict N-myristoylated proteins from any completed genome [46,48], these bioinformatics and predictive approaches have several drawbacks. Most notably, using known N-myristoylated motifs from various organisms to inform our identification of N-myristoylated proteins in *T. cruzi* may well lead to difficulties, since previous studies have shown a degree of variability in this motif across different organisms [49–51]. With this in mind, work is underway to identify directly the N-myristoylated proteins comprising the *T. cruzi* N-myristoylome using a click chemistry approach.

In conclusion, we have demonstrated that NMT from *T. cruzi* is both an essential and druggable target. However, discovery of more potent and selective inhibitors will be required to achieve a suitable therapeutic window for the treatment of Chagas’ disease.

## References

[1] Schmunis G. A. (2007). Epidemiology of Chagas disease in non-endemic countries: the role of international migration. Mem. Inst. Oswaldo Cruz.

[2] Rassi A., Rassi A., Marin-Neto J. A. (2010). Chagas disease. Lancet.

[3] World Health Organization (2012). Chagas disease (American trypanosomiasis): fact sheet (revised in August 2012). Wkly Epidemiol. Rec..

[4] WHO Expert Committee (2002). Control of Chagas disease. World Health Organ. Tech. Rep. Ser..

[5] Cancado J. R. (2002). Long term evaluation of etiological treatment of Chagas disease with benznidazole. Rev. Inst. Med. Trop. Sao Paulo.

[6] Jackson Y., Alirol E., Getaz L., Wolff H., Combescure C., Chappuis F. (2010). Tolerance and safety of nifurtimox in patients with chronic Chagas disease. Clin. Infect. Dis..

[7] Hasslocher-Moreno A. M., do Brasil P. E., de Sousa A. S., Xavier S. S., Chambela M. C., Sperandio da Silva G. M. (2012). Safety of benznidazole use in the treatment of chronic Chagas’ disease. J. Antimicrob. Chemother..

[8] Towler D. A., Eubanks S. R., Towery D. S., Adams S. P., Glaser L. (1987). Amino-terminal processing of proteins by *N*-myristoylation. Substrate specificity of *N*-myristoyl transferase. J. Biol. Chem..

[9] Towler D. A., Adams S. P., Eubanks S. R., Towery D. S., Jackson-Machelski E., Glaser L., Gordon J. I. (1987). Purification and characterization of yeast myristoyl CoA:protein *N*-myristoyltransferase. Proc. Natl. Acad. Sci. U.S.A..

[10] Lodge J. K., Jackson-Machelski E., Toffaletti D. L., Perfect J. R., Gordon J. I. (1994). Targeted gene replacement demonstrates that myristoyl-CoA:protein *N*-myristoyltransferase is essential for viability of *Cryptococcus neoformans*. Proc. Natl. Acad. Sci. U.S.A..

[11] Knoll L. J., Levy M. A., Stahl P. D., Gordon J. I. (1992). Analysis of the compartmentalization of myristoyl-CoA:protein *N*-myristoyltransferase in *Saccharomyces cerevisiae*. J. Biol. Chem..

[12] Price H. P., Menon M. R., Panethymitaki C., Goulding D., McKean P. G., Smith D. F. (2003). Myristoyl-CoA: protein *N*-myristoyltransferase, an essential enzyme and potential drug target in kinetoplastid parasites. J. Biol. Chem..

[13] Frearson J. A., Brand S., McElroy S. P., Cleghorn L. A. T., Smid O., Stojanovski L., Price H. P., Guther M. L. S., Torrie L. S., Robinson D. A. (2010). *N*-myristoyltransferase inhibitors as new leads to treat sleeping sickness. Nature.

[14] Bhatnagar R. S., Futterer K., Farazi T. A., Korolev S., Murray C. L., Jackson-Machelski E., Gokel G. W., Gordon J. I., Waksman G. (1998). Structure of N-myristoyltransferase with bound myristoylCoA and peptide substrate analogs. Nat. Struct. Biol..

[15] Glover C. J., Hartman K. D., Felsted R. L. (1997). Human *N*-myristoyltransferase amino-terminal domain involved in targeting the enzyme to the ribosomal subcellular fraction. J. Biol. Chem..

[16] Godsel L. M., Engman D. M. (1999). Flagellar protein localization mediated by a calcium-myristoyl/palmitoyl switch mechanism. EMBO J..

[17] Hunter K. J., Le Quesne S. A., Fairlamb A. H. (1994). Identification and biosynthesis of *N*^1^,*N*^9^-*bis*(glutathionyl)aminopropylcadaverine (homotrypanothione) in *Trypanosoma cruzi*. Eur. J. Biochem..

[18] Silveira F. T., Viana Dias M. G., Pereira Pardal P., Oliveira Lobâo A., Britto Melo G. (1979). Nono caso-autóctone de doença de Chagas registrado no estado do Pará, Brasil (Nota prévia). Hiléia Médica.

[19] Ariyanayagam M. R., Oza S. L., Mehlert A., Fairlamb A. H. (2003). Bis(glutathionyl)spermine and other novel trypanothione analogues in *Trypanosoma cruzi*. J. Biol. Chem..

[20] Ammerman N. C., Beier-Sexton M., Azad A. F. (2008). Growth and maintenance of Vero cell lines. Curr. Protoc. Microbiol..

[21] Franzen O., Ochaya S., Sherwood E., Lewis M. D., Llewellyn M. S., Miles M. A., Andersson B. (2011). Shotgun sequencing analysis of *Trypanosoma cruzi* I Sylvio X10/1 and comparison with *T. cruzi* VI CL Brener. PLoS Negl. Trop. Dis..

[22] Studier F. W. (2005). Protein production by auto-induction in high-density shaking cultures. Protein Expr. Purif..

[23] Martin K., Smith T. K. (2005). The myo-inositol-1-phosphate synthase gene is essential in *Trypanosoma brucei*. Biochem. Soc. Trans..

[24] Vazquez M. P., Levin M. J. (1999). Functional analysis of the intergenic regions of TcP2 β gene *loci* allowed the construction of an improved *Trypanosoma cruzi* expression vector. Gene.

[25] Xu D., Brandan C. P., Basombrio M. A., Tarleton R. L. (2009). Evaluation of high efficiency gene knockout strategies for *Trypanosoma cruzi*. BMC Microbiol..

[25a] Marques A., Nakayasu E., Almeida I. (2011). Purification of extracellular and intracellular amastigotes of *Trypanosoma cruzi* from mammalian host-infected cells. Protoc. Exch..

[26] Tovar J., Fairlamb A. H. (1996). Extrachromosomal, homologous expression of trypanothione reductase and its complementary mRNA in *Trypanosoma cruzi*. Nucleic Acids Res..

[27] Wyllie S., Oza S. L., Patterson S., Spinks D., Thompson S., Fairlamb A. H. (2009). Dissecting the essentiality of the bifunctional trypanothione synthetase-amidase in *Trypanosoma brucei* using chemical and genetic methods. Mol. Microbiol..

[28] Boisson B., Meinnel T. (2003). A continuous assay of myristoyl-CoA:protein *N*-myristoyltransferase for proteomic analysis. Anal. Biochem..

[29] Panethymitaki C., Bowyer P. W., Price H. P., Leatherbarrow R. J., Brown K. A., Smith D. F. (2006). Characterization and selective inhibition of myristoyl-CoA: protein *N*-myristoyltransferase from *Trypanosoma brucei* and *Leishmania major*. Biochem. J..

[30] Cruz A. K., Titus R., Beverley S. M. (1993). Plasticity in chromosome number and testing of essential genes in *Leishmania* by targeting. Proc. Natl. Acad. Sci. U.S.A..

[31] Rohloff P., Rodrigues C. O., Docampo R. (2003). Regulatory volume decrease in *Trypanosoma cruzi* involves amino acid efflux and changes in intracellular calcium. Mol. Biochem. Parasitol..

[32] Goncalves V., Brannigan J. A., Thinon E., Olaleye T. O., Serwa R., Lanzarone S., Wilkinson A. J., Tate E. W., Leatherbarrow R. J. (2012). A fluorescence-based assay for *N*-myristoyltransferase activity. Anal. Biochem..

[33] Rampoldi F., Sandhoff R., Owen R. W., Grone H. J., Porubsky S. (2012). A new, robust, and nonradioactive approach for exploring *N*-myristoylation. J. Lipid Res..

[34] Hertz-Fowler C., Ersfeld K., Gull K. (2001). CAP5.5, a life-cycle-regulated, cytoskeleton-associated protein is a member of a novel family of calpain-related proteins in *Trypanosoma brucei*. Mol. Biochem. Parasitol..

[35] Weinburg R. A., McWherter C. A., Freeman K. S., Wood D. C., Gordon J. I., Lee S. C. (1995). Genetic studies reveal that myristoylCoA-protein *N*-myristoyltransferase is an essential enzyme in *Candida albicans*. Mol. Microbiol..

[36] Yang S. H., Shrivastav A., Kisinski C., Sharma R. K., Chen M. H., Berthiaume L. G., Peters L. L., Chuang P. T., Young S. G., Bergo M. O. (2005). *N*-myristoyltransferase 1 is essential in early mouse development. J. Biol. Chem..

[37] Martins V. D., Okura M., Maric D., Engman D. M., Vieira M., Docampo R., Moreno S. N. J. (2010). Acylation-dependent export of *Trypanosoma cruzi* phosphoinositide-specific phospholipase C to the outer surface of amastigotes. J. Biol. Chem..

[38] Lodge J. K., Jackson-Machelski E., Devadas B., Zupec M. E., Getman D. P., Kishore N., Freeman S. K., McWherter C. A., Sikorski J. A., Gordon J. I. (1997). *N*-myristoylation of Arf proteins in *Candida albicans*: an *in vivo* assay for evaluating antifungal inhibitors of myristoyl-CoA: protein *N*-myristoyltransferase. Microbiology.

[39] Bell A. S., Mills J. E., Williams G. P., Brannigan J. A., Wilkinson A. J., Parkinson T., Leatherbarrow R. J., Tate E. W., Holder A. A., Smith D. F. (2012). Selective inhibitors of protozoan protein *N*-myristoyltransferases as starting points for tropical disease medicinal chemistry programs. PLoS Negl. Trop. Dis..

[40] Goncalves V., Brannigan J. A., Whalley D., Ansell K. H., Saxty B., Holder A. A., Wilkinson A. J., Tate E. W., Leatherbarrow R. J. (2012). Discovery of *Plasmodium vivax N*-myristoyltransferase inhibitors: screening, synthesis, and structural characterization of their binding mode. J. Med. Chem..

[41] Price H. P., Guther M. L., Ferguson M. A., Smith D. F. (2010). Myristoyl-CoA:protein *N*-myristoyltransferase depletion in trypanosomes causes avirulence and endocytic defects. Mol. Biochem. Parasitol..

[42] Price H. P., Stark M., Smith D. F. (2007). *Trypanosoma brucei* ARF1 plays a central role in endocytosis and Golgi-lysosome trafficking. Mol. Biol. Cell.

[43] Engstler M., Thilo L., Weise F., Grunfelder C. G., Schwarz H., Boshart M., Overath P. (2004). Kinetics of endocytosis and recycling of the GPI-anchored variant surface glycoprotein in *Trypanosoma brucei*. J. Cell Sci..

[44] Allen C. L., Goulding D., Field M. C. (2003). Clathrin-mediated endocytosis is essential in *Trypanosoma brucei*. EMBO J..

[45] Porto-Carreiro I., Attias M., Miranda K., de Souza W., Cunha-e-Silva N. (2000). *Trypanosoma cruzi* epimastigote endocytic pathway: cargo enters the cytostome and passes through an early endosomal network before storage in reservosomes. Eur. J. Cell Biol..

[46] Mills E., Price H. P., Johner A., Emerson J. E., Smith D. F. (2007). Kinetoplastid PPEF phosphatases: dual acylated proteins expressed in the endomembrane system of *Leishmania*. Mol. Biochem. Parasitol..

[47] Cordero E. M., Nakayasu E. S., Gentil L. G., Yoshida N., Almeida I. C., da Silveira J. F. (2009). Proteomic analysis of detergent-solubilized membrane proteins from insect-developmental forms of *Trypanosoma cruzi*. J. Proteome Res..

[48] Maurer-Stroh S., Eisenhaber B., Eisenhaber F. (2002). N-terminal *N*-myristoylation of proteins: prediction of substrate proteins from amino acid sequence. J. Mol. Biol..

[49] Traverso J. A., Giglione C., Meinnel T. (2013). High-throughput profiling of *N*-myristoylation substrate specificity across species including pathogens. Proteomics.

[50] Towler D. A., Adams S. P., Eubanks S. R., Towery D. S., Jackson-Machelski E., Glaser L., Gordon J. I. (1988). Myristoyl CoA:protein *N*-myristoyltransferase activities from rat liver and yeast possess overlapping yet distinct peptide substrate specificities. J. Biol. Chem..

[51] Rocque W. J., McWherter C. A., Wood D. C., Gordon J. I. (1993). A comparative analysis of the kinetic mechanism and peptide substrate specificity of human and *Saccharomyces cerevisiae* myristoyl-CoA:protein *N*-myristoyltransferase. J. Biol. Chem..

